# Identifying Protein Phosphorylation Sites with Kinase Substrate Specificity on Human Viruses

**DOI:** 10.1371/journal.pone.0040694

**Published:** 2012-07-23

**Authors:** Neil Arvin Bretaña, Cheng-Tsung Lu, Chiu-Yun Chiang, Min-Gang Su, Kai-Yao Huang, Tzong-Yi Lee, Shun-Long Weng

**Affiliations:** 1 Department of Computer Science and Engineering, Yuan Ze University, Chung-Li, Taiwan; 2 Graduate Program in Biomedical Informatics, Yuan Ze University, Chung-Li, Taiwan; 3 Department of Obstetrics and Gynecology, Hsinchu Mackay Memorial Hospital, Hsinchu, Taiwan; 4 Mackay Medicine, Nursing and Management College, Taipei, Taiwan; 5 Department of Medicine, Mackay Medical College, New Taipei City, Taiwan; 6 Department of Biological Science and Technology, National Chiao Tung University, Hsinchu, Taiwan; 7 Institute of Bioinformatics and Systems Biology, National Chiao Tung University, Hsinchu, Taiwan; University of Queensland, Australia

## Abstract

Viruses infect humans and progress inside the body leading to various diseases and complications. The phosphorylation of viral proteins catalyzed by host kinases plays crucial regulatory roles in enhancing replication and inhibition of normal host-cell functions. Due to its biological importance, there is a desire to identify the protein phosphorylation sites on human viruses. However, the use of mass spectrometry-based experiments is proven to be expensive and labor-intensive. Furthermore, previous studies which have identified phosphorylation sites in human viruses do not include the investigation of the responsible kinases. Thus, we are motivated to propose a new method to identify protein phosphorylation sites with its kinase substrate specificity on human viruses. The experimentally verified phosphorylation data were extracted from virPTM – a database containing 301 experimentally verified phosphorylation data on 104 human kinase-phosphorylated virus proteins. In an attempt to investigate kinase substrate specificities in viral protein phosphorylation sites, maximal dependence decomposition (MDD) is employed to cluster a large set of phosphorylation data into subgroups containing significantly conserved motifs. The experimental human phosphorylation sites are collected from Phospho.ELM, grouped according to its kinase annotation, and compared with the virus MDD clusters. This investigation identifies human kinases such as CK2, PKB, CDK, and MAPK as potential kinases for catalyzing virus protein substrates as confirmed by published literature. Profile hidden Markov model is then applied to learn a predictive model for each subgroup. A five-fold cross validation evaluation on the MDD-clustered HMMs yields an average accuracy of 84.93% for Serine, and 78.05% for Threonine. Furthermore, an independent testing data collected from UniProtKB and Phospho.ELM is used to make a comparison of predictive performance on three popular kinase-specific phosphorylation site prediction tools. In the independent testing, the high sensitivity and specificity of the proposed method demonstrate the predictive effectiveness of the identified substrate motifs and the importance of investigating potential kinases for viral protein phosphorylation sites.

## Introduction

Viruses are biological agents that interrupt and manipulate normal cellular functions [1], [2]. Viruses infect humans and progress inside the body leading to various diseases and complications. An increasing number of human viruses has been recorded and studied over the years, such as the human immunodeficiency virus (HIV) and the human herpes virus (HHV) [3]. Most viruses interact with host-cell proteins in order to gain control of cellular machinery. By perturbing the cellular regulatory networks, these viruses interfere with the normal cellular processes, such as cell growth and gene expression [4]. It has been reported that viruses have evolved to use the process of phosphorylation by host-cell kinases as a means of enhancing replication and inhibition of normal cellular functions [5].

Protein phosphorylation is the most widespread and well-studied post-translational modification (PTM) in eukaryotic cells [6], [7]. The process involves the transfer of a phosphate group by a protein kinase to a target protein substrate – commonly on serine (S), threonine (T), and tyrosine (Y) residues [8]. Protein kinases recognize short linear motifs for initiating phosphorylation. These linear motif signatures are shown to be vital in further investigating kinase-substrate interactions [9], [10]. Short linear motif signatures found in phosphorylated virus proteins can be used to further elucidate interactions between host-cell kinase and virus protein substrates. Although not yet clearly elucidated, these interactions are linked to viral progression in the human body.

Further understanding of viral protein phosphorylation is essential due to its importance with regard to viral progression. However, there is a great deal of difficulty in experimentally identifying viral protein phosphorylation sites using mass spectrometry-based techniques; thus, computational methods for identifying protein phosphorylation sites have been proposed. Existing phosphorylation site prediction tools can be classified into three categories: general or non-specific, organism-specific, and kinase-specific [11]. Computational tools built to predict non-specific phosphorylation sites such as NetPhos [12] are usually trained using all available experimentally-verified phosphorylation data regardless of organism information. However, phosphorylation patterns may not be exactly the same for all organisms. With this, organism-specific phosphorylation site predictors were developed. Following its initial version, NetPhos was retrained using phosphorylation sites from yeast proteins and bacterial proteins, respectively, resulting to NetPhosYeast [13] and NetPhosBac [14]. These tools are among the first phosphorylation predictors that identifies phosphorylation sites according to a specific organism. A plant-specific phosphorylation prediction tool, PhosPhAt 3.0 [15], was developed using phosphorylation data from Arabidopsis Thaliana as its training data for identifying phosphorylation sites specific to the Arabidopsis Thaliana species. A previous work was done which utilizes scan-X [16] to identify phosphorylation sites on viral proteins [17]; however, it has not investigated the various substrate motifs for viral protein phosphorylation sites.

In phosphorylation, it is known that substrates are targeted by kinases according to a specific pattern. Specific amino acid residues at certain positions of a protein greatly affect the specificity of a particular kinase [18]. Because of this, kinase-specific phosphorylation site predictors have been developed. NetPhosK [19], which utilizes a neural network method, is able to predict phosphorylation sites for 18 kinases including cAMP-dependent protein kinase, protein kinase C, caseine kinase II, and calmodulin-dependent protein kinase II. ScanSite [20] utilizes an entropy approach to match a predicted phosphorylation site according to a motif. It covers 65 eukaryotic protein kinases including casein kinase I, casein kinase II, calmodulin-dependent kinase II, extracellular signal regulated kinase 1, and protein kinase A. KinasePhos [21], [22] incorporates support vector machine (SVM) with a sequence-based amino acid coupling-pattern analysis to identify phosphorylation sites for 29 S kinases, 16 T kinases, and 26 Y kinases. PPSP [23] adapts a Bayesian decision theory approach in order to predict phosphorylation sites for 68 protein kinase groups. GPS [24] classifies 408 protein kinases according to a four-level hierarchy and predicts phosphorylation sites according to this classification. NetPhorest [25] utilizes artificial neural networks and position-specific scoring matrices in order to build a linear motif atlas for phosphorylation networks. NetPhorest is also able to probabilistically classify experimentally identified phosphorylation sites according to the 179 kinases that it currently covers. With most of the existing kinase-specific phosphorylation site prediction tools requiring prior knowledge of experimentally verified substrates and its kinase, a method is developed to be able to predict kinase-specific phosphorylation sites based solely on protein sequence [18]. Predikin [26] is a method that first demonstrated the application of structure-based information for the prediction of phosphorylation sites in proteins. The method utilized by Predikin identifies significant residues from a given query sequence and associates it with a particular kinase specificity in order to predict phosphorylation sites for a certain kinase [26].

Based on the current state of research, there is still a lack of understanding as to what kind of host kinases specifically phosphorylates viral proteins. Therefore, we are motivated to develop a method to investigate the substrate motifs and identify potential host kinases for viral protein phosphorylation sites. The identification of kinases is deemed important as these are heavily pursued pharmaceutical targets due to their mechanism role in various diseases [27]. Moreover, identifying kinases responsible for phosphorylation would be beneficial for selective inhibition therapies and the development of kinase inhibitors for treatment. This work presents a method for identifying potential human kinases for viral phosphorylation sites. Literature is surveyed to support the identified potential human kinases. To further evaluate the method, the kinase substrate motifs were utilized to construct predictive models for identifying phosphorylation sites on viral proteins.

## Results and Discussion

### Data Collection and Statistics

**Figure 1:**
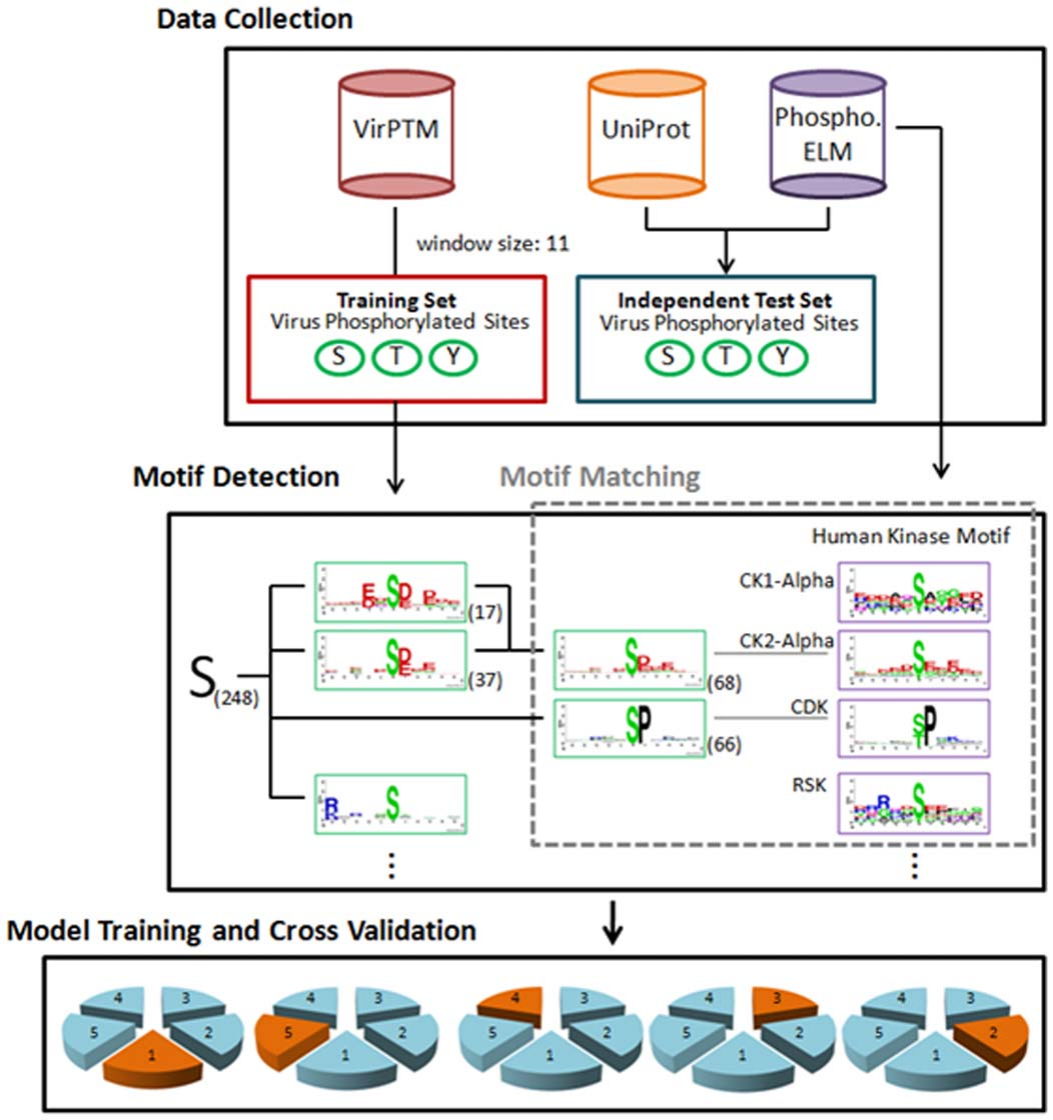
Analytical flowchart. The proposed method involves three major steps: data collection, motif detection, and model training and cross validation.

Figure 1 presents the analytical flowchart of this study which comprises of three major steps - data collection, motif detection and motif matching, and model training and cross-validation. For this study, viral protein phosphorylation data in humans are collected from virPTM [17], UniProtKB [28], and Phospho.ELM [29]. In order to maintain the genuineness of the data set, only literature-based viral protein phosphorylation data are collected from virPTM version 1.0 which contains 329 experimentally verified phosphorylation data on 111 virus proteins (47 virus types), as the distribution of virus phosphorylation data shown in Figure S1. As this study aims to analyze human kinases that phosphorylate virus proteins, virPTM entries annotated as phosphorylated by virus kinases are disregarded. This resulted in 233, 54, and 14 phosphorylated S, T, and Y sites from 104 virus proteins as shown in Table S1. A set of viral protein phosphorylation data are also collected from UniProtKB version 2011_01_11 containing 525997 protein records. Experimentally verified viral protein phosphorylation data in humans are obtained by filtering out entries annotated as “by similarity”, “potential”, and “probable” resulting in 57 phosphorylation data on 23 human virus proteins. The collected data is further refined by removing entries annotated as phosphorylated by virally-encoded kinases resulting in 43, and 12 phosphorylated S, and T sites from 22 virus proteins as shown in Table S1. Another set of viral protein phosphorylation data are collected from Phospho.ELM version 0910 containing 42575 phosphorylated protein entries from 47 species. Experimentally verified viral protein phosphorylation data in humans are obtained by extracting entries annotated as LTP which represents data that have been identified by using low-throughput processes. As shown in Table S1, this resulted in 7, and 2 phosphorylated S, and Y sites from 6 proteins with no data annotated as phosphorylated by a virus kinase.

In order to investigate the residues surrounding the phosphorylation sites, sequence fragments are extracted using a window size of 11 centered on S, T, and Y. A window size of 11 consists of 11 amino acid residues placed from position −5 to 5. Fragments having a phosphorylated residue on position 0 are obtained and regarded as positive data while fragments centered on non-phosphorylated residues are regarded as negative data. As shown in Table 1, 233, 54, and 14 positive S, T, and Y fragments as well as 2588, 1170, and 65 S, T, and Y negative fragments are obtained from virPTM. From the UniProt dataset, 24, and 10 positive S and T fragments are obtained as well as 217, and 159 negative S and T fragments. Furthermore, two positive S and Y fragments as well as 67, and 16 negative S and Y fragments are obtained from the Phospho.ELM dataset. With reference to PlantPhos [30], a smaller number of negative fragments are obtained to match the number of positive fragments. The *K*-means clustering method [31], [32] is employed for acquiring a subset that represents the whole negative data set. The value of *K* which denotes the number of samples to be obtained from the negative set is defined by the number of corresponding positive data. This resulted in an equal number of positive and negative S, T, and Y fragments respectively in the three data sets as shown in Table 1. Finally, the balanced non-redundant data from virPTM is regarded as the training set, while the balanced non-redundant data from UniProt and Phospho.ELM are regarded as the independent testing set.

**Table 1:** Statistics of data used for this study.

Data Set	Source	Residue	Type	DataCount	Balanced Data
**Training Set**	**virPTM**	**S**	Positive	233	233
			Negative	2588	233
		**T**	Positive	54	54
			Negative	1170	54
		**Y**	Positive	14	14
			Negative	65	65
**Independent**	**UniProtKB**	**S**	Positive	24	24
**Testing Set**			Negative	217	24
		**T**	Positive	10	10
			Negative	159	10
	**Phospho.ELM**	**S**	Positive	2	2
			Negative	67	2
		**Y**	Positive	2	2
			Negative	16	2

### Investigation of Kinase Substrate Motifs

It is observed that the phosphorylated sequences in each subgroup clustered using maximal dependence decomposition (MDD) show a conserved motif representing its substrate site specificity. The flanking amino acids (−5 ∼ +5) of the non-redundant phosphorylation sites, which are centered on position 0, are graphically visualized as sequence logos using WebLogo. Maximal dependence decomposition is executed multiple times with varying values in order to obtain the most optimal minimum cluster size. Setting the minimum cluster size to 50 for pSer data yielded 7 clusters as shown in Table S2. Increasing the minimum cluster size did not result in any clusters and further lowering of the minimum cluster size resulted in several similar clusters; therefore, the minimum cluster size is set to 50. After MDD, further refinement is done by analyzing these groups through its corresponding entropy plots. It is observed that some groups contain very similar motifs, some show no conserved motif, and some groups have too little data which makes the motif unreliable. Some of these groups are further combined together and visualized using WebLogo. For the resulting pSer MDD clusters, S1 and S2 which show very similar motifs are combined into S1 as shown in Table S3. Also, cluster S5 which shows a weak conserved motif is combined with cluster S6 to form a new cluster S4 as shown in Table S3. For organization, the remaining clusters are renamed accordingly.

For virus pThr and pTyr data, the minimum cluster size is set to ten. Similar to the process of selecting the minimum cluster size for pSer, increasing the minimum cluster size did not result in any clusters and further lowering of the minimum cluster size resulted in several similar clusters. This resulted in three clusters in pThr as shown in Table S4, and five clusters in Y as shown in Table S5. However, due to the very low number of pTyr data, the resulting MDD clusters show no conserved motif and contain very few fragments to be considered reliable. Therefore, for this study, pTyr is not further clustered using MDD prior to training a pTyr model.

**Figure 2:**
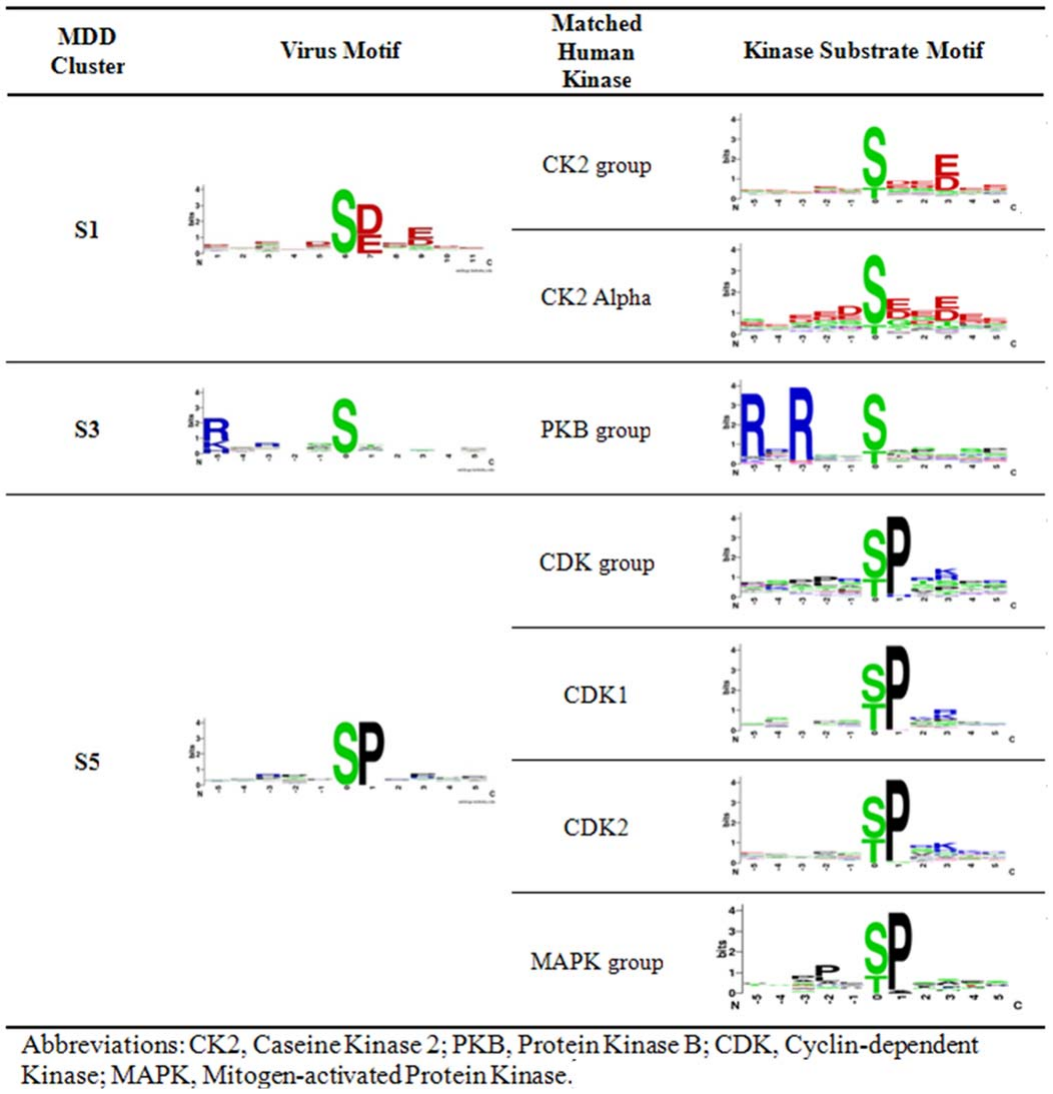
pSer virus motif – human motif matches.

**Figure 3:**
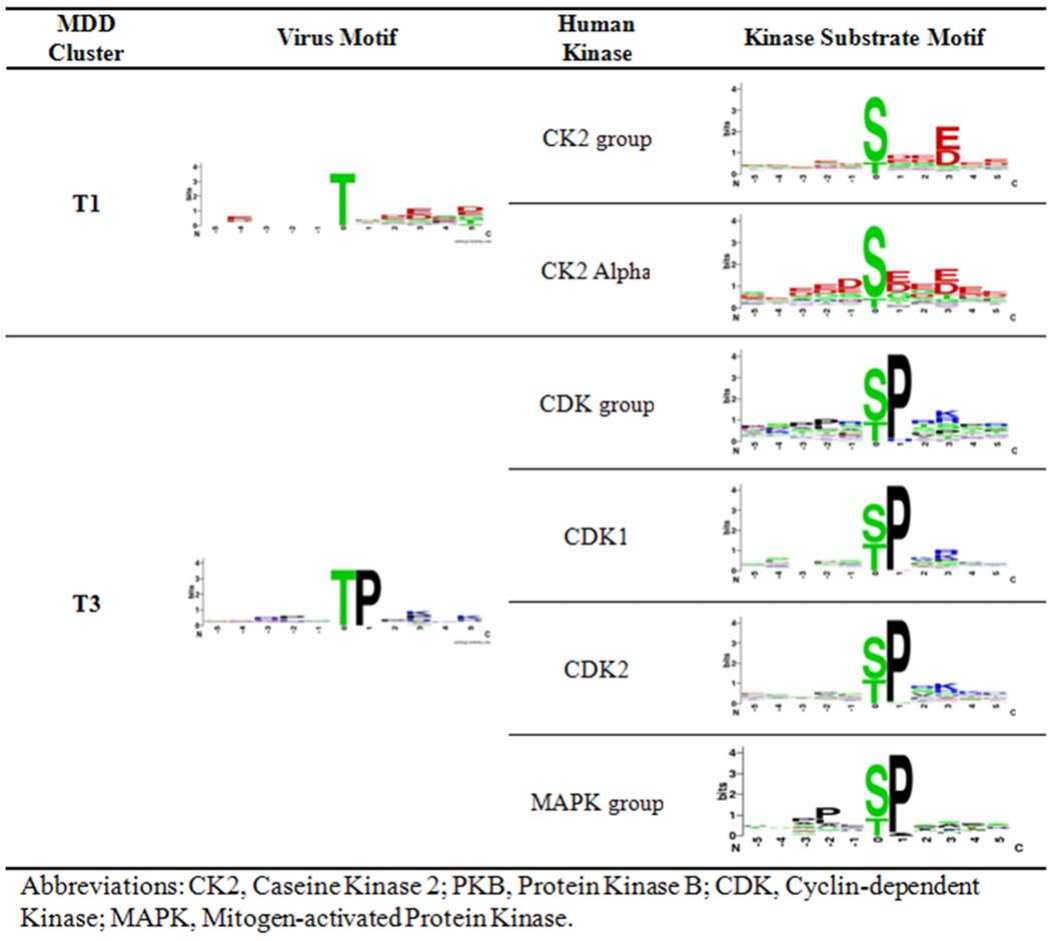
pThr virus motif – human motif matches.

In order to identify potential host kinases for human virus substrates, the motif of each MDD-generated viral protein phosphorylation cluster is compared with the discovered human kinase substrate specificities. As shown in Figure 2, cluster S1 is matched to be potentially phosphorylated by caseine kinase 2 (CK2) group and CK2 alpha due to a strong similarity with regard to the conserved aspartic acid and glutamic acid residues in positions +1, and +3. Protein kinase B (PKB) group is also matched to be a potential host kinase that phosphorylates virus proteins in cluster S3 due to a similarly conserved arginine residue at position -5. Furthermore, cluster S5 is matched to be potentially phosphorylated by cyclin-dependent kinase (CDK) group, CDK1, CDK2, and mitogen-activated protein kinase (MAPK) group due to a conserved proline in position +1 as shown in its respective motifs. In terms of pThr, cluster T1 is matched to be potentially phosphorylated by CK2 group and CK2 alpha due to a similarly conserved aspartic acid and glutamic acid residues in position +3. Cluster T3 is then matched to be potentially phosphorylated by CDK group, CDK1, CDK2, MAPK group due to a conserved proline in position +1 as shown in Figure 3.

Further analyzing the matched motifs, a literature survey is done in order to find studies that experimentally identify human kinases which phosphorylate specific virus protein substrates. Previous studies [33], [34] show that CK2 group phosphorylates hepatitis C virus (HCV) NS5A proteins and HIV-1 gp120, gp41, p27, and p17 proteins to name a few, on both S and T residues. These findings support the matching of MDD groups S1 and T1 with CK2 group. CK2 family phosphorylates various proteins which are associated with the viral infection of HCV, HIV, HSV, HBV and HPV [35], [36]. With regard to PKB which is matched with cluster S3, it is reported to be involved in the regulation of the herpes simplex virus (HSV) 1 [37]. Experimental research also claims that PKB signaling benefits coxsackie virus B3 replication [38]. Although it is unclear whether PKB directly phosphorylates a virus protein, the match between MDD group S3 and the substrate specificity of PKB group suggests a phosphorylation interaction between the said kinase and some virus protein substrates. Reports have also been published that CDK, particularly CDK2, is involved in the transcription and replication of HIV-1 by means of phosphorylation [39], [40]. Also, it is reported that CDK mediates phosphorylation of the human influenza A virus on T-215 of the NS1 protein [41]. Furthermore, a previous study [42] identifies CDK1 as the human kinase responsible for phosphorylating varicella-zoster virus (VZV), commonly known as the chickenpox virus, on S224 of the IE63 protein.

To demonstrate the effectiveness of MDD clustering method, the MDD-detected motifs are compared with two well-known motif discover tools, Motif-X [43] and MoDL [44]. Tables S6 and S7 show that MDD could identify new motifs for viral protein phosphorylation sites and is comparable to other methods. As shown in Table S6, MDD is able to detect five motifs from the available virus S phosphorylation data. From these five motifs, three are supported by previous literature. It should be noted that Motif-X failed to detect the virus pSer motif with conserved R amino acid residue at position -5, matched with PKB group. Moreover, Motif-X was only able to detect three motifs for virus pSer sites with two motifs having similar amino acid conservations (D and E at positions +1 and +3). With regard to virus pThr sites, MDD was able to detect three motifs with two of these being supported by literature. On the other hand, Motif-X is also able to detect the virus T motif with conserved E residue at position +3, which is matched with CK2 group. As for the MDD and MoDL, the two methods produce similar phosphorylation motifs as shown in Table S7.

### Cross-validation of Identifying Viral Protein Phosphorylation Sites with Kinase Substrate Motifs

The cross-validation process includes the selection of the threshold parameter for each model. The threshold parameter is a specific bit score that serves as the cutoff value of HMMsearch for determining matching query sequences for an HMM [45]. With reference to a previous work [22], [30], the threshold is selected by first testing each value from the range of −20 to 0 as the bit score. The threshold is tuned to a specific value which allows an HMM to yield a high and balanced specificity and sensitivity for a specific HMM. Table 2 shows the threshold score selected for each model of pSer together with its individual predictive performance and the predictive performance of using all models together. Furthermore, Table 3 shows the threshold score selected for each model of pThr together with its individual predictive performance and the predictive performance of using all models together. It can be observed that MDD clusters featuring an obvious conserved motif are able to yield a higher predictive accuracy as compared to those showing no conserved motif. For instance, cluster S1 which features an observed aspartic acid and glutamic acid residues in positions +1, and +3 yields an accuracy of 93.4% when used individually. On the other hand, MDD clusters that do not seem to have an obvious conserved motif yield a significantly lower predictive performance. For instance, cluster T2 which does not show a strongly conserved motif based on its entropy plot only yields an accuracy of a 46.6% when used individually.

**Table 2:** Five-Fold Cross Validation Results on Serine MDD-Clustered HMMs.

Group	Number of positive data	HMMER bit score	Pre	Sn	Sp	Acc
**S1**	54	−11	93.1%	94.1%	92.7%	93.4%
**S2**	34	−11	80.0%	94.2%	76.6%	85.4%
**S3**	20	−9	84.3%	90.0%	80.0%	85.0%
**S4**	59	−8	66.4%	74.6%	60.6%	67.6%
**S5**	66	−10	89.3%	98.4%	87.6%	93.0%
**Combined Performance**			**82.7%**	**90.3%**	**79.5%**	**84.9%**

Abbreviations: Pre, precision; Sn, sensitivity; Sp, specificity; Acc, accuracy.

**Table 3:** Five-Fold Cross Validation Results on Threonine MDD-Clustered HMMs.

Group	Number of positive data	HMMER bit score	Pre	Sn	Sp	Acc
**T1**	19	−10	92.0%	100%	90.0%	95%
**T2**	16	−11	43.3%	50.0%	43.3%	46.6%
**T3**	19	−10	95.0%	90.0%	95.0%	92.5%
**Combined Performance**			**76.8%**	**80.0%**	**76.1%**	**78.0%**

Abbreviations: Pre, precision; Sn, sensitivity; Sp, specificity; Acc, accuracy.

**Figure 4:**
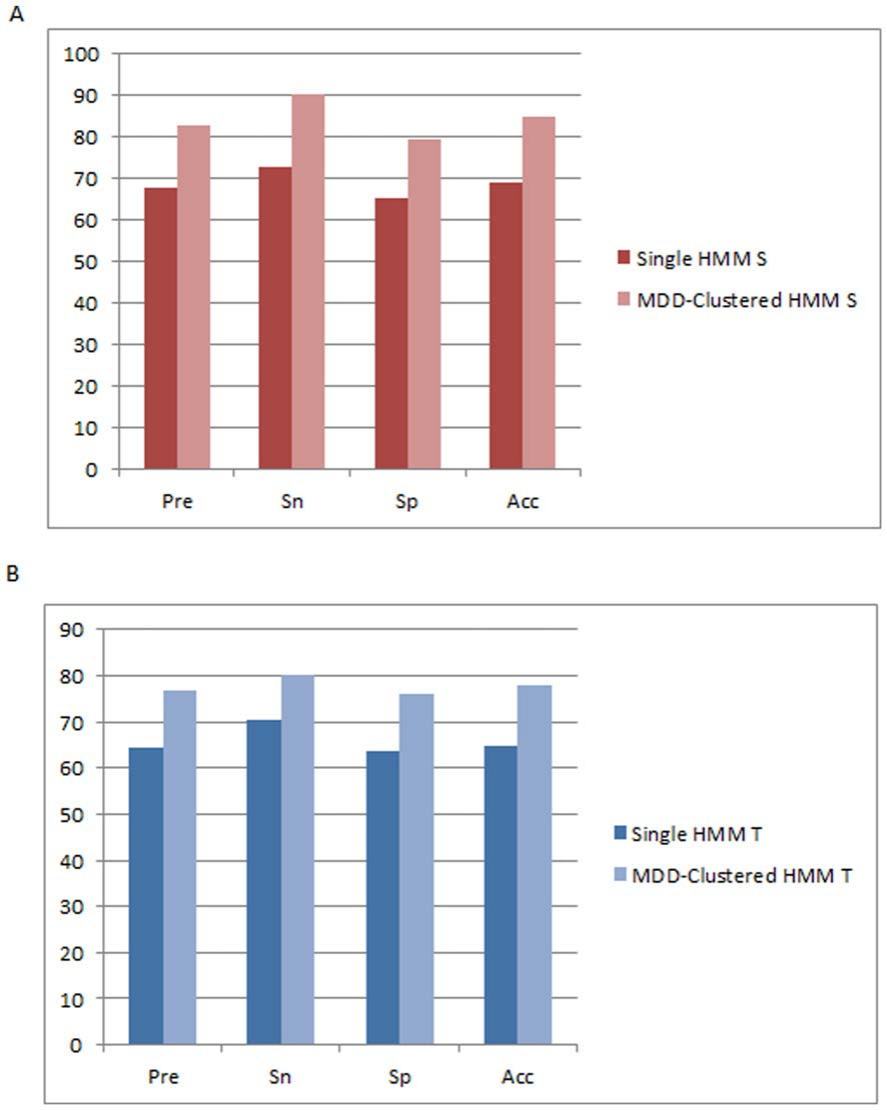
Comparison of five-fold cross validation performance. (**A**) Comparison of 5-fold cross validation results between an S HMM which does not utilize prior MDD-clustering and S HMMs which utilize prior MDD-clustering. (**B**) Comparison of 5-fold cross validation results between a T HMM which does not utilize prior MDD-clustering and T HMMs which utilize prior MDD-clustering.

According to a five-fold cross-validation evaluation, the predictive performance of MDD-clustered HMM performs significantly better than non-MDD clustered HMM of pSer, and pThr. As shown in Figure 4A, S HMMs which utilize prior MDD clustering yields a higher performance with a precision rate of 82.70%, a sensitivity rate of 90.30%, a specificity rate of 79.50%, and an accuracy rate of 84.90% as compared to a non-MDD clustered S HMM which yields a precision rate of 67.80%, a sensitivity rate of 72.90%, a specificity rate of 65.20%, and an accuracy rate of 69.00%. On the other hand, T HMMs which utilizes prior MDD clustering yields a higher performance with a precision rate of 76.8%, a sensitivity rate of 80.0%, a specificity rate of 76.1%, and an accuracy rate of 78.1% as compared to a non-MDD clustered T HMMs which yields a precision rate of 64.5%, a sensitivity rate of 70.3%, a specificity rate of 63.6%, and an accuracy rate of 64.9% as shown in Figure 4B. Due to a lack of virus pTyr data, MDD clustering could not be performed to form HMMs for computationally identifying pTyr sites; thus, a single HMM is used for pTyr until sufficient experimentally-verified virus pTyr sites are acquired.

### Independent Testing

**Figure 5:**
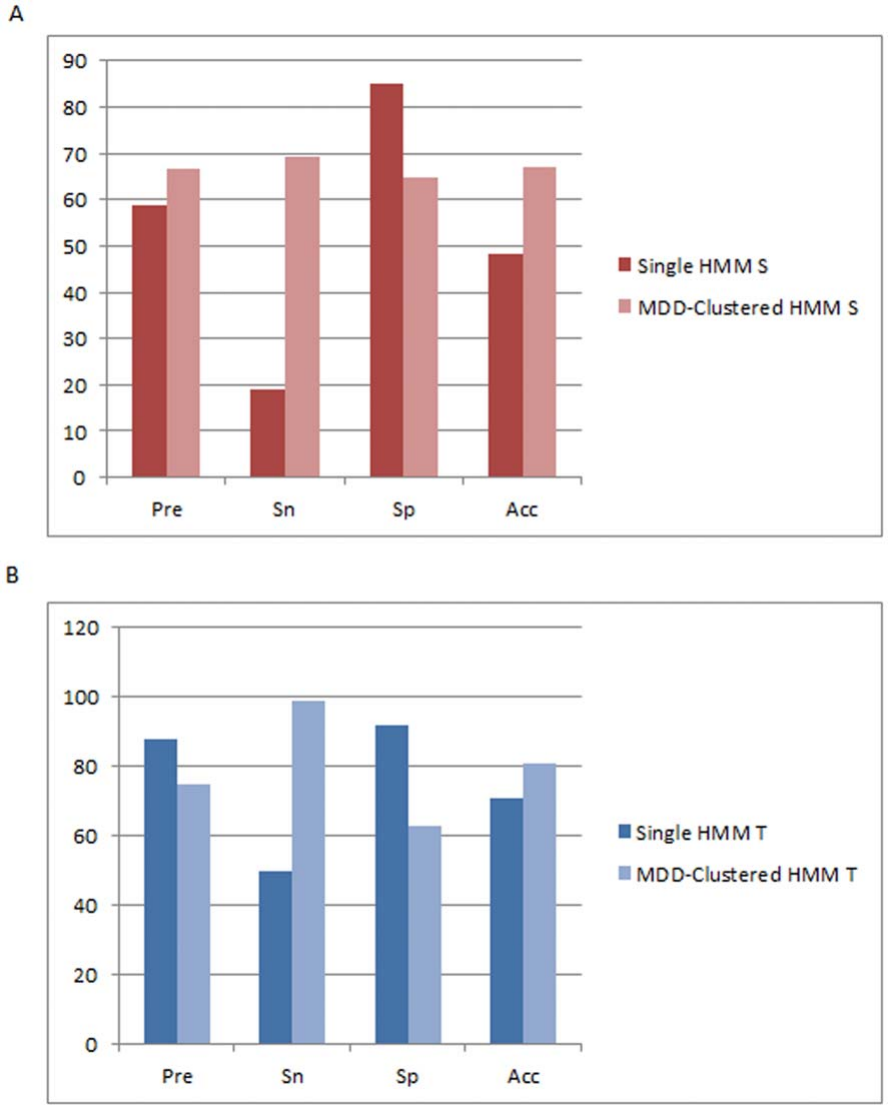
Comparison of independent testing performance. (**A**) Comparison of independent test results between an S HMM which does not utilize prior MDD-clustering and S HMMs which utilize prior MDD-clustering. (**B**) Comparison of independent test results between a T HMM which does not utilize prior MDD-clustering and T HMMs which utilize prior MDD-clustering.

An independent test is done due to the possibility of an over-fit of the models in the training set which may lead to an overestimation of its predictive performance [30]. The data set obtained from both UniProtKB and Phospho.ELM. As shown in Table 4, each individual MDD-clustered S HMM yields an average of 70.70% precision, 19.23% sensitivity, 90.31% specificity, and 54.76% accuracy. Furthermore, using all the S MDD-clustered HMMs altogether yields a precision rate of 66.66%, a sensitivity rate of 69.23%, a specificity rate of 64.91%, and an accuracy rate of 66.92% which is significantly higher as compared to the performance of a non-MDD clustered S HMM as shown in Figure 5A. On the other hand, Table 5 shows that using the independent data on each MDD-clustered T HMM yields an average of 71.44% precision, 36.67% sensitivity, 84.00% specificity, and 60.33% accuracy. Furthermore, using all the T MDD-clustered HMMs altogether yields a precision rate of 74.96%, a sensitivity rate of 99.00%, a specificity rate of 62.70%, and an accuracy rate of 80.85% which is significantly higher and more balanced as compared to the performance of a non-MDD clustered T HMM as shown in Figure 5B.

**Table 4:** Independent Test Results of Serine MDD-clustered HMMs.

Residue	MDDgroup	Threshold	Pre	Sn	Sp	Acc
	**S1**	−11	89.5%	11.5%	98.1%	54.8%
	**S2**	−11	65.3%	34.6%	80.0%	57.3%
**S**	**S3**	−7	58.6%	11.5%	90.8%	51.2%
	**S4**	−8	67.5%	11.5%	93.5%	52.5%
	**S5**	−10	72.6%	26.9%	89.2%	58.1%
**Combined** **Performance**			66.7%	69.2%	64.6%	66.9%

Abbreviations: Pre, precision; Sn, sensitivity; Sp, specificity; Acc, accuracy.

**Table 5:** Independent Test Results of Threonine MDD-clustered HMMs.

Residue	MDDgroup	Threshold	Pre	Sn	Sp	Acc
	**T1**	−10	42.5%	20.0%	71.0%	45.5%
**T**	**T2**	−6	88.4%	50.0%	92.0%	71.0%
	**T3**	−10	80.7%	40.0%	89.0%	64.5%
**Combined** **Performance**			75.0%	99.0%	62.7%	80.9%

Abbreviations: Pre, precision; Sn, sensitivity; Sp, specificity; Acc, accuracy.

In order to further evaluate our approach, each predicted phosphorylation site resulting from the independent test is studied. A survey on existing literature is done by referencing UniProt [28] in order to find relevant literature that will support the phosphorylation of a predicted site as well as its identified potential kinase. Table 6 lists down each predicted phosphorylation site together with its predicted kinase and supporting literature, if any. Three sites predicted to be phosphorylated by specific host kinases agree with reports from literature. HIV-1 protein P05923 which is predicted to be phosphorylated by CK2 at S56 matched with the findings of a previous study [46] that experimentally identified CK2 as the catalytic kinase of P05923 at S56. Moreover, human T-lymphotrophic virus (HTLV) 1 proteins P03345 and P03409 which were both predicted to be phosphorylated by CDK at S105 and S336, respectively, matched with a report [47] that, although does not confirm phosphorylation, points out the relation of CDK to HTLV-1 protein replication. Seven sites predicted to be phosphorylated by specific host kinases are reported to be phosphorylated by yet to be known human kinases. HTLV-1 protein P0C205 and human respiratory syncytial virus (HRSV) protein P12579 were all predicted to be phosphorylated by model S2 at positions S70, S116, and S161, respectively. Interestingly, these sites are reported by previous studies [28], [48] to be phosphorylated by host, but the kinase remains unknown. Moreover, some sites which have been reported to be phosphorylated by a yet to be known host kinase are identified with a potential specific kinase by our method such as CDK for HIV-1 protein P69718 at position S99. Six sites predicted to be phosphorylated by specific host kinases are reported to be phosphorylated by another kinase. Three of these sites belong to HHV-4 protein P03191 which is reported to be phosphorylated by a virally-encoded kinase [49]. Some sites, however, have been previously identified to be phosphorylated by a human kinase, such as extracellular signal-regulated kinase (ERK) for human papillomavirus (HPV) 16 protein P06922 at T57 [50] but is predicted by our method to be phosphorylated by CDK. This suggests that the potential host kinases identified in our method could provide new leads with regard to virus substrate- host kinase investigations. Twenty sites predicted to be phosphorylated by specific host kinases had no records of the responsible kinase in literature. This suggests that further investigation could be focused on the potential kinases identified by our method in order to experimentally verify host kinases for specific phosphorylation sites.

**Table 6:** Summary of predicted phosphorylation sites on human viruses.

Virus Name	Protein ID	Position	Predicted Kinase	Literature-annotated Kinase	Reference
HHV-5	P18139	S462	CK2; CK2 Alpha; Model S2	Unknown	
HIV-1	P05923	S56	CK2; CK2 Alpha; Model S2	CK2	[46]
HTLV-1	P0C205	S70	Model S2	By Host(Unknown)	[58]
HIV-1	P05923	S52	Model S2	CK2	[28]
HRSV	P12579	S116	Model S2	By Host(Unknown)	[28]
HHV-4	P03191	S305	Model S2	Unknown	
HRSV	P12579	S161	Model S2	By Host (Unknown)	[48]
HTLV-1	P03345	S105	Model S2; PKB; CDK; MAPK	MAPK1; CDK*	[47], [59]
HHV-3	P09258	S343	CDK; MAPK; Model S2	Unknown	
HIV-1	P69723	S144	PKB	Unknown	
HTLV-1	P0C205	S165	PKB	Unknown	
HTLV-1	P03409	S336	PKB; CDK; MAPK	CDK*	[47]
HRSV	P12579	S117	PKB	By Host(Unknown);	[48]
HIV-1	P05928	S79	Model S4	By Host(Unknown)	[60], [61]
HHV-5	P69332	S338	Model S4	Unknown	
HTLV-1	P0C205	S177	Model S4	Unknown	
HTLV-1	P0C205	S147	Model S4	Unknown	
HIV-1	P05928	S94	Model S4	By Host (Unknown)	[60], [61]
HTLV-1	P0C205	S97	CDK; MAPK	Unknown	
HHV-4	P03191	S337	CDK; MAPK	Viral BGLF4 kinase	[49]
HIV-1	P69718	S99	CDK; MAPK	By Host (Unknown)	[2]
HHV-4	P03191	S349	CDK; MAPK	Viral BGLF4 kinase	[49]
HTLV-1	P0C205	S177	CDK; MAPK	Unknown	
HHV-4	P03191	S121	CDK; MAPK	Unknown	
HTLV-1	P0C205	T174	CK2; CK2 Alpha	By Host (Unknown)	[58]
HHV-4	P03191	T344	CK2; CK2 Alpha; CDK; MAPK	Viral BGLF4 kinase	[49]
HPV-16	P06922	T71	CK2; CK2 Alpha	Unknown	
HTLV-1	P03409	T242	CK2; CK2 Alpha	Unknown	
HTLV-1	P03409	T48	Model T2	Unknown	
HIV-1	P69723	T188	Model T2	Unknown	
HTLV-1	P03409	T215	Model T2	Unknown	
HTLV-1	P0C205	T174	Model T2	Unknown	
HTLV-1	P03409	T322	Model T2	Unknown	
HHV-1	P06437	T313	Model T2	Unknown	
HIV-1	P69723	T155	CDK; MAPK	Unknown	
HHV-4	P03191	T355	CDK; MAPK	Viral BGLF4 kinase	[49]
HPV-16	P06922	T57	CDK; MAPK	ERK	[50]

The summaries of human viruses and kinases are presented in Table S9 and S10, respectively.

* Relation between human kinase and virus protein reported in literature.

### Comparison with Other Phosphorylation Site Prediction Tools

To further demonstrate the effectiveness of the proposed method, the independent testing data is used to make a comparison between the performances of three popular kinase-specific phosphorylation site prediction tools, Predikin 2.0 [26], KinasePhos 2.0 [21], and GPS 2.1 [51]. According to the collection of experimentally verified protein phosphorylation data from UniProtKB and Phospho.ELM, a total of 36 viral protein phosphorylation sites (in 28 viral protein sequences), which are not included in the training data, are regarded as the positive set of the independent testing data. In order to evaluate the predictive specificity, the S and T residues, which are not annotated as the phosphorylation sites in the 28 viral protein sequences, are regarded as the negative set of the independent testing data. As a result, the independent testing data consisting of 36 positive sites and 392 negative sites are used to compare the predictive precision, sensitivity, specificity and accuracy between the MDD-clustered HMMs, Predikin 2.0, KinasePhos 2.0, and GPS 2.1. Without any prior information of catalytic kinases for the testing data, all of the kinase-specific models in the prediction tools are chosen for predicting the phosphorylation sites. Table 7 indicates that all of the prediction tools containing multiple models have a high predictive sensitivity. However, it is notable that the MDD-clustered HMMs are able to yield a higher specificity compared to the other tools. Since potential kinase family information for viral protein phosphorylation sites are still unknown, Predikin yields a higher specificity than KinasePhos and GPS. Overall, the proposed method outperforms the other three tools. With reference to the comparison of independent testing, the high sensitivity and specificity of MDD-clustered HMMs present the importance of investigating kinase substrate motifs for viral protein phosphorylation sites.

**Table 7:** Comparison of independent testing performance with other kinase-specific phosphorylation site prediction tools.

Tools	MDD-clustered HMMs	PREDIKIN 2.0	KinasePhos 2.0	GPS 2.1
Number of true positive predictions	36	33	36	36
Number of false positive predictions	89	145	172	189
Number of true negative predictions	303	247	220	203
Number of false negative predictions	0	3	0	0
Precision	28.9%	18.5%	17.3%	16.0%
Sensitivity	100.0%	91.7%	100.0%	100.0%
Specificity	77.3%	63.1%	56.1%	51.8%
Accuracy	79.2%	65.4%	59.8%	55.8%

### Conclusions

In this study, viral protein phosphorylation sites found in humans are further elucidated by means of identifying their potential catalytic human kinase. The study is done using experimentally verified viral protein phosphorylation sites obtained from virPTM [17]. This study explores the use of short linear motifs to further identify viral protein phosphorylation sites. MDD is employed to detect kinase substrate motifs on viral protein phosphorylation sites. Based on the detected viral protein phosphorylation motifs, potential host kinases are identified according to their motif signatures. Finally, profile hidden Markov models (HMMs) are trained in order to predict viral protein phosphorylation sites according to host kinase motifs. Our approach has identified human kinases such as CK2, PKB, CDK, and MAPK as potential catalytic kinases for virus protein substrates. A five-fold cross validation evaluation shows that our method can identify viral protein phosphorylation sites based on the identified phosphorylation motifs on human viruses. Furthermore, an independent test done using data not included in the model training confirms the ability of our MDD-clustered HMMs.

In addition to the consideration of linear sequence motifs, substrate recruitment is very important in the investigation of kinase substrate specificity. However, with limited information regarding kinase-specific phosphorylation sites on viral proteins, the substrate recruitment of kinases could not be investigated for the viral protein phosphorylation data. This is the main reason why this work develops a computational method to investigate potential kinase substrate motifs for viral protein phosphorylation sequences. The approach offers the scientific community clues regarding human kinases that may be responsible for the phosphorylation of human virus proteins. It is important to note, however, that the further acquisition of experimentally verified viral protein phosphorylation sites is required to identify more meaningful viral protein phosphorylation motifs. Also, a more abundant set of experimentally verified kinase-annotated human phosphorylation sites could be used to improve the collection of substrate motifs. These developments could benefit our method by allowing the identification of more potential human kinases catalyzing virus proteins.

## Materials and Methods

### Data Construction

In this work, the experimentally verified data of viral protein phosphorylation sites are collected from virPTM [17], UniProtKB [28], and Phospho.ELM [29]. In order to avoid the acquisition of overlapping phosphorylation data from the three databases, each data obtained from one database is compared to the data obtained from the other two databases based on their position and UniProtKB accession number. If the same data is found in two or more datasets, only one record is retained and the redundant data is removed. As shown in Table S1, this method resulted in 24 phosphorylated S (pSer), and 10 phosphorylated T (pThr) from UniProtKB, and 2 pSer, and 2 phosphorylated Y (pTyr) from Phospho.ELM. Since the number of negative fragments is much greater than the number of corresponding positive fragments, the data is not balanced. With reference to PlantPhos [30], a smaller number of negative fragments are obtained by the *K*-means clustering method [31], [32] which is employed for acquiring a subset that represents the whole negative data set. A data point which has a minimal distance from other data points surrounding it is selected as a representative data. For this study, *K*-means clustering is performed based on sequence identity. The value of *K* which denotes the number of samples to be obtained from the negative set is defined by the number of corresponding positive data.

### Motif Detection and Comparison

The phosphorylated fragments from the obtained training set are used to investigate the motif signatures of phosphorylated virus proteins. In order to explore the conserved motifs from a large data set, MDD is applied to cluster all phosphorylated fragments into subgroups that show statistically significant motifs. MDD is a methodology that groups a set of aligned signal sequences to moderate a large group into subgroups that capture the most significant dependencies between positions. Previous studies [30], [32] have proposed the grouping of protein sequences into smaller groups prior to creating prediction models. For this study, MDD is applied using MDDLogo [32]. MDD adopts chi-square test to evaluate the dependence of amino acid occurrence between two positions, Ai and Aj, which surround the phosphorylation site. In order to extract motifs that have conserved biochemical property of amino acids when doing MDD, we categorize the twenty types of amino acids into five groups: neutral, acid, basic, aromatic, and imino groups, as shown in Table S8. A contingency table of the amino acids occurrence between two positions is then constructed, as presented in Figure S2. The chi-square test is defined as:
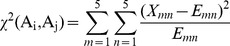
(1)where *Xmn* represents the number of sequences that have the amino acids of group m in position *Ai* and have the amino acids of group n in position *Aj*, for each pair (*Ai, Aj*) with *i*≠*j*. *Emn* is calculated as 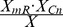, where *X_mR_*  =  *X_m1_*+ …+*X_m5_*, *X_Cn_*  =  *X_1n_*+ …+*X_5n_*, and *X* denotes the total number of sequences. If a strong dependence is detected (defined as *X^2^* that is larger than 34.3, corresponding to a cutoff level of *P* = 0.005 with 16 degrees of freedom) between two positions, then the process is continued as described by Burge and Karlin [52]. As illustrated in Figure S2, it can be observed that position +1 has the maximal dependence with the occurrence of imino amino acids. Subsequently, all data can be divided into two subgroups where one has the occurrence of imino amino acids in position +1 and the other not having an occurrence of imino amino acids in position +1. MDD clustering is a recursive process which divides the positive set into tree-like subgroups. When applying MDD to cluster the sequences in the positive set, a parameter, i.e., the minimum-cluster-size, should be set. If the size of a subgroup is less than the minimum-cluster-size, the subgroup will not be divided any further. The MDD process terminates until all the subgroup sizes are less than the value of the minimum-cluster-size. With reference to previous works that utilize MDD [21], [30], [32], [53], there exists no set values for the parameters of MDD clustering. In order to obtain an optimal minimum cluster size, MDD clustering is executed using various values. Each subgroup is represented using WebLogo [54] to graphically visualize the corresponding substrate motif. The resulting clusters are then analyzed as to whether or not they contain significant conserved motifs. Subgroups with very similar motifs are further grouped together into a single cluster in order to provide more meaningful groups and avoid redundant clusters as shown in the motif detection step in Figure 1.

Meanwhile, in order to identify the various human kinase substrate specificities, human phosphorylated proteins annotated with their catalytic kinases are collected from Phospho.ELM. The phosphorylation sites are extracted using a window size of 11 and are grouped together according to its annotated human kinase. Each human kinase group is then graphically visualized as sequence logos using WebLogo. The motifs of the MDD-generated viral protein phosphorylation clusters and the visualized substrate specificity of human kinases are compared. A substrate-kinase match is selected by comparing the conservation of amino acids in each position (−5 ∼ +5) appearing as obvious motifs in the visualized sequence logos of each virus MDD clusters and human kinase. Fragments of amino acids are extracted from MDD clusters and human kinase groups using a window length of 2*n+*1 that is centered on phosphorylation sites. Next, a positional weighted matrix (PWM) [55] is adopted to represent the relative frequency of amino acids around the phosphorylation sites. A matrix of *(*2*n+*1*)*×*m* elements is used to represent each MDD-cluster or kinase group, where 2*n+*1 stands for the window length and *m* consists of 21 elements for the 20 types of amino acids and for one terminal signal. Then, the Euclidean distance [56] is applied to measure the matrix similarity between MDD clusters and kinase groups. As the scoring calculation by Euclidean distance, the smaller distance value has a higher similarity between MDD cluster and kinase group. Finally, for each MDD cluster, the most similar kinase group is regarded as the matched host kinase and the sequence logo is visualized for verification.

### Model Training and Cross-validation

In this work, profile HMM is built from the site sequences of each MDD-clustered subgroup. An HMM describes a probability distribution over a potentially infinite number of sequences [45]. It can also be used to detect distant relationships between amino acids sequences. Here, the software package HMMER version 2.3.2 [45] is used to build profile HMMs, to calibrate the HMMs, and to search the putative phosphorylation sites against the protein sequences. HMM builds a model based on positive instances of a class; thus, in this study, only positive data are utilized to build a predictive model. After the application of MDD clustering on viral protein phosphorylation data, each of the MDD-clustered subgroups is taken as a training set to build a profile HMM.

For each model of the MDD-clustered subgroups, a threshold parameter is selected as a cut-off value in identifying potential positive data from a query [45]. An optimized threshold is selected as the value which gives the most optimal cross-validation performance for each training model. To search the hits of a HMM, HMMER returns both a bit score and an expectation value (E-value). The bit score is the base two logarithm of the ratio between the probability that the query sequence is a significant match and the probability that it is generated by a random model. The E-value represents the expected number of sequences with a score greater than or equal to the returned HMMER bit scores. A search result with an HMMER bit score greater than the threshold parameter is taken as a positive prediction. While decreasing the bit score threshold favors finding true positives, increasing the bit score threshold favors finding true negatives. Therefore, the threshold must be set to obtain a balanced number of true positives and true negatives.

Prior to the construction of a final model, the predictive performance of the models with varying parameters are evaluated by performing k-fold cross validation. In doing k-fold cross validation, the training data is divided into k groups by splitting each dataset into approximately equal sized subgroups. In one round of cross-validation, a subgroup is regarded as the test set, and the remaining k-1 subgroups are regarded as the training set. The cross-validation process is repeated k rounds, with each of the k subgroups used as the test set in turn. Then, the k results are combined to produce a single estimation. The advantage of k-fold cross-validation is that all original data are regarded as both training set and test set, and each data is used for testing exactly once [57]. In this study, k is set to five. The models are initially evaluated using five-fold cross-validation and are gauged by measuring their predictive performance. The following measures of predictive performance are defined as:
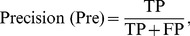
(1)

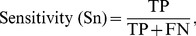
(2)

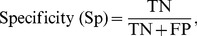
(3)

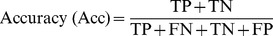
(4)where TP, TN, FP and FN represent the numbers of true positives, true negatives, false positives and false negatives, respectively. Subsequent to the construction of the predictive model, an independent test using the data set obtained from both UniProtKB and Phospho.ELM is carried out to further evaluate the predictive performance of each HMM.

## Supporting Information

Figure S1 **Distribution of the collected viral protein phosphorylation data.**
(TIF)

Figure S2 **The analytical flowchart of MDD.**
(TIF)

Table S1 **Statistics of experimentally verified phosphorylation sites from virPTM, UniProtKB, and Phospho.ELM.**
(DOC)

Table S2 **pSer Virus MDD-clustered Motifs.**
(DOC)

Table S3 **Refined pSer Virus MDD-clustered Motifs.**
(DOC)

Table S4 **pThr Virus MDD-clustered Motifs.**
(DOCX)

Table S5 **pTyr Virus MDD-clustered Motifs.**
(DOCX)

Table S6 **Comparison of pSer and pThr motifs between MDD clustering and Motif-X.**
(DOCX)

Table S7 **Comparison of pSer and pThr motifs between MDD clustering and MoDL.**
(DOCX)

Table S8 **The amino acids group used in MDD clustering.**
(DOCX)

Table S9 **Summary of Human Viruses.**
(DOCX)

Table S10 **Summary of Human Kinases.**
(DOCX)
