# Supplementary figures and images for: Identifying Protein Phosphorylation Sites with Kinase Substrate Specificity on Human Viruses
Source: PLoS One. 2012 Jul 23;7(7):e40694. doi: 10.1371/journal.pone.0040694 (PMC3402495; doi:10.1371/journal.pone.0040694)

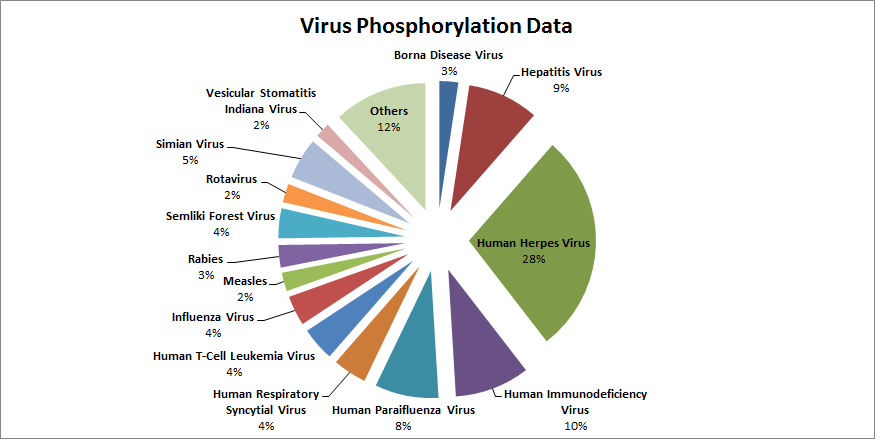

Supplement: Figure S1 — Distribution of the collected viral protein phosphorylation data. (TIF) [file pone.0040694.s001.tif]

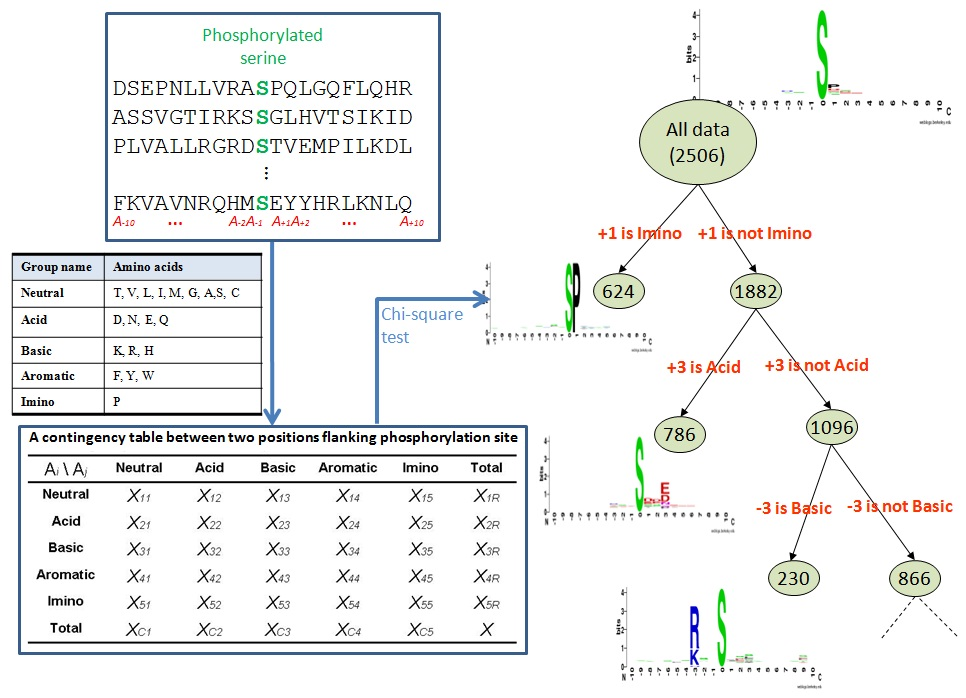

Supplement: Figure S2 — The analytical flowchart of MDD. (TIF) [file pone.0040694.s002.tif]
