# Supplementary material for: Identifying Protein Phosphorylation Sites with Kinase Substrate Specificity on Human Viruses
Source: PLoS One. 2012 Jul 23;7(7):e40694. doi: 10.1371/journal.pone.0040694 (PMC3402495; doi:10.1371/journal.pone.0040694)
Supplement: Table S1 — Statistics of experimentally verified phosphorylation sites from virPTM, UniProtKB, and Phospho.ELM. (DOC) [file pone.0040694.s003.doc]

**Supplementary Table S1**. Statistics of experimentally verified phosphorylation sites from virPTM, UniProtKB, and Phospho.ELM

| **Data source** | | **Phosphorylated Proteins** | **pSer** | **pThr** | **pTyr** |
| --- | --- | --- | --- | --- | --- |
| **virPTM**  v.1 | Original | 104 | 233 | 54 | 14 |
|  | Non-Redundant | 104 | 233 | 54 | 14 |
| **UniProtKB**  2011_01_11 | Original | 22 | 43 | 12 | 0 |
|  | Non-Redundant | 12 | 24 | 10 | 0 |
| **Phospho.ELM**  0910 | Original | 6 | 7 | 0 | 2 |
|  | Non-Redundant | 4 | 2 | 0 | 2 |
