# Supplementary material for: Identifying Protein Phosphorylation Sites with Kinase Substrate Specificity on Human Viruses
Source: PLoS One. 2012 Jul 23;7(7):e40694. doi: 10.1371/journal.pone.0040694 (PMC3402495; doi:10.1371/journal.pone.0040694)
Supplement: Table S3 — Refined pSer Virus MDD-clustered Motifs. (DOC) [file pone.0040694.s005.doc]

**Supplementary Table S3**. Refined pSer Virus MDD-clustered Motifs

| **Residue** | **MDD Cluster** | **Motif** | **Number of Fragments** |
| --- | --- | --- | --- |
| **Serine** | **S1** | 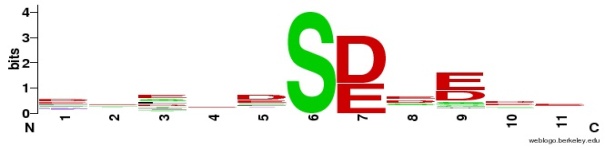 | 54 |
|  | **S2** | 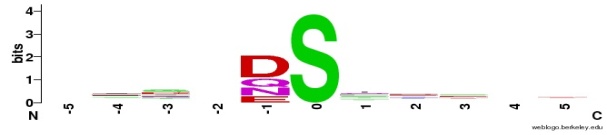 | 34 |
|  | **S3** | 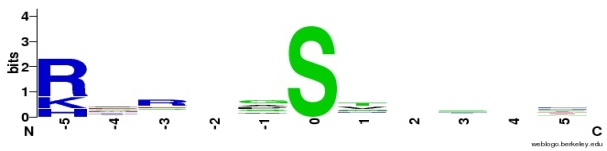 | 20 |
|  | **S4** | 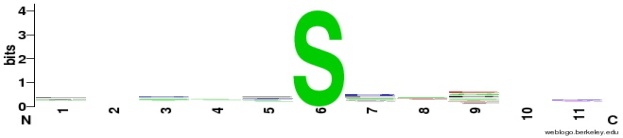 | 59 |
|  | **S5** | 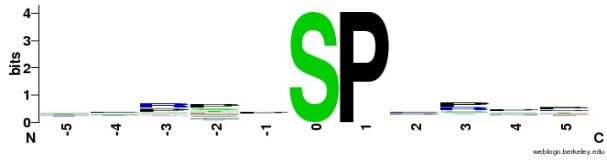 | 66 |
