# Supplementary material for: Identifying Protein Phosphorylation Sites with Kinase Substrate Specificity on Human Viruses
Source: PLoS One. 2012 Jul 23;7(7):e40694. doi: 10.1371/journal.pone.0040694 (PMC3402495; doi:10.1371/journal.pone.0040694)
Supplement: Table S4 — pThr Virus MDD-clustered Motifs. (DOCX) [file pone.0040694.s006.docx]

**Supplementary Table S4**. pThr Virus MDD-clustered Motifs

| **Residue** | **MDD Cluster** | **Motif** | **Number of Fragments** |
| --- | --- | --- | --- |
| **Threonine** | **T1** | 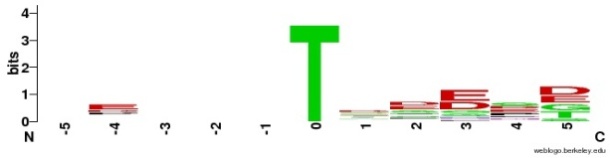 | 19 |
|  | **T2** | 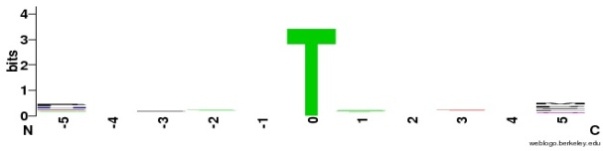 | 16 |
|  | **T3** | 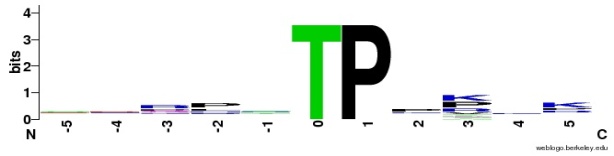 | 19 |
