# Supplementary material for: Identifying Protein Phosphorylation Sites with Kinase Substrate Specificity on Human Viruses
Source: PLoS One. 2012 Jul 23;7(7):e40694. doi: 10.1371/journal.pone.0040694 (PMC3402495; doi:10.1371/journal.pone.0040694)
Supplement: Table S5 — pTyr Virus MDD-clustered Motifs. (DOCX) [file pone.0040694.s007.docx]

**Supplementary Table S5**. pTyr Virus MDD-clustered Motifs

| **Residue** | **MDD Cluster** | **Motif** | **Number of Fragments** |
| --- | --- | --- | --- |
| **Tyrosine** | **Y1** | 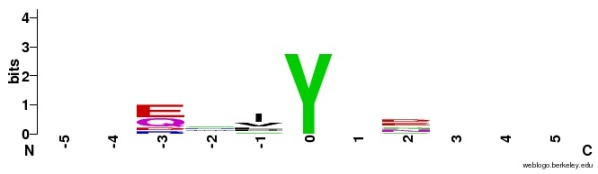 | 9 |
|  | **Y2** | 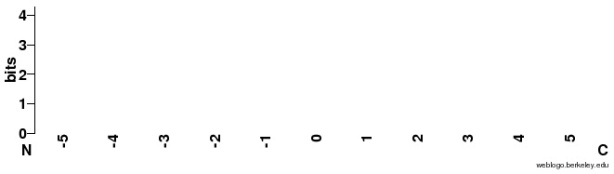 | 1 |
|  | **Y3** | 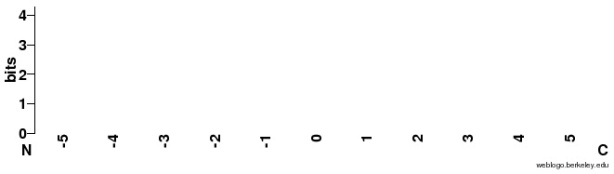 | 1 |
|  | **Y4** | 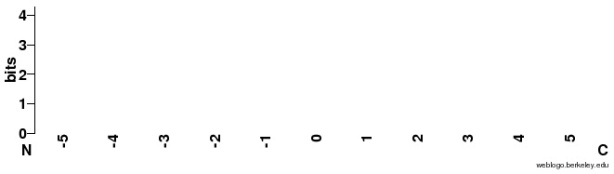 | 3 |
