# Supplementary material for: Identifying Protein Phosphorylation Sites with Kinase Substrate Specificity on Human Viruses
Source: PLoS One. 2012 Jul 23;7(7):e40694. doi: 10.1371/journal.pone.0040694 (PMC3402495; doi:10.1371/journal.pone.0040694)
Supplement: Table S6 — Comparison of pSer and pThr motifs between MDD clustering and Motif-X. (DOCX) [file pone.0040694.s008.docx]

**Supplementary Table S6**. Comparison of pSer and pThr motifs between MDD clustering and Motif-X.

| **MDD Clustering** | | **Motif-X** | |
| --- | --- | --- | --- |
| **MDD-detected Motif** | **Number of Fragments** | **Motif-X Motif** | **Number of Fragments** |
| 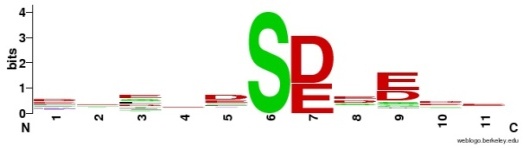 | 54 | 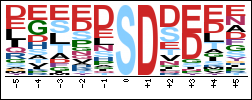 | 33 |
|  |  | 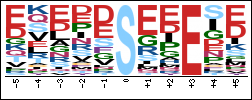 | 26 |
| 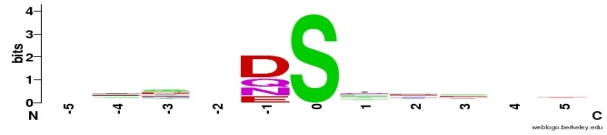 | 34 |  |  |
| 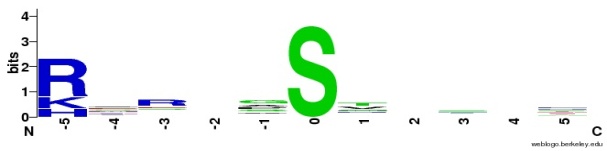 | 20 |  |  |
| 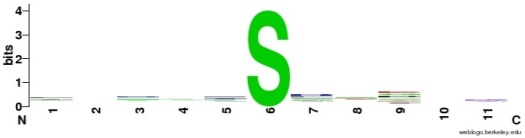 | 59 |  |  |
| 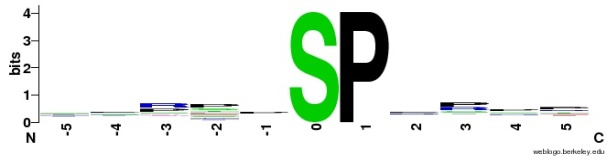 | 66 | 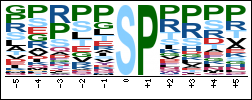 | 64 |
| 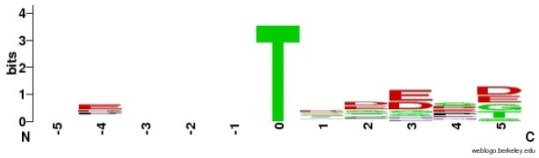 | 19 | 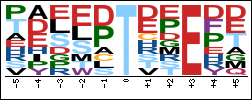 | 10 |
| 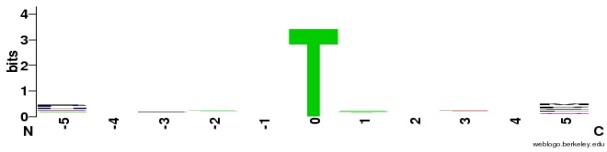 | 16 |  |  |
| 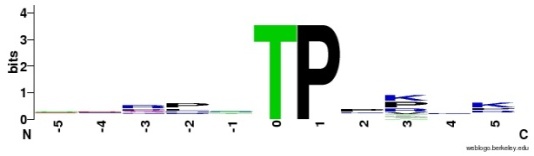 | 19 | 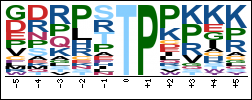 | 19 |
