# Supplementary material for: Identifying Protein Phosphorylation Sites with Kinase Substrate Specificity on Human Viruses
Source: PLoS One. 2012 Jul 23;7(7):e40694. doi: 10.1371/journal.pone.0040694 (PMC3402495; doi:10.1371/journal.pone.0040694)
Supplement: Table S7 — Comparison of pSer and pThr motifs between MDD clustering and MoDL. (DOCX) [file pone.0040694.s009.docx]

**Supplementary Table S7**. Comparison of pSer and pThr motifs between MDD clustering and MoDL.

| **MDD Clustering** | | **MoDL** | |
| --- | --- | --- | --- |
| **MDD-detected Motif** | **Number of Fragments** | **MoDL Motif** | **Number of Fragments** |
| 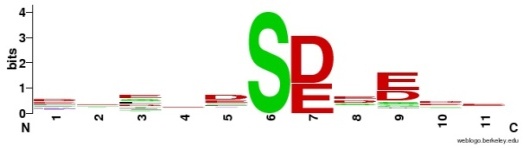 | 54 | ......S[DE]..... | 54 |
| 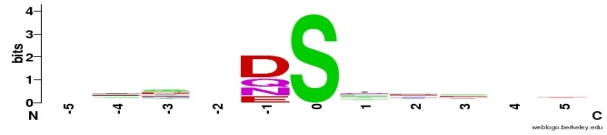 | 34 | .....[DE]S...... | 21 |
| 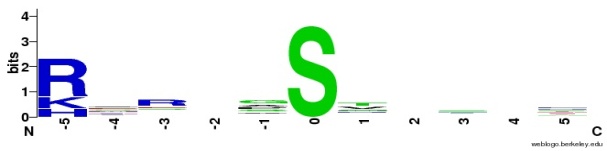 | 20 | .[RKH]....S...... | 20 |
| 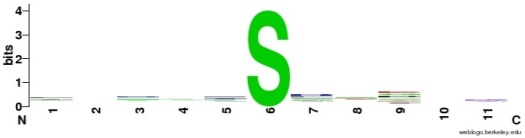 | 59 |  |  |
| 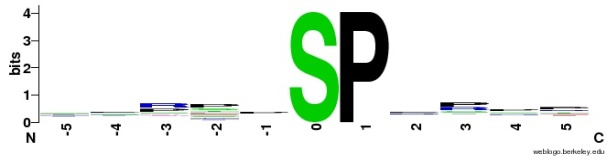 | 66 | ......SP..... | 66 |
| 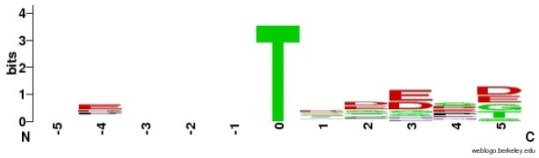 | 19 | ......T..[DE]... | 15 |
| 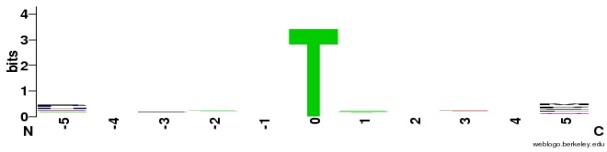 | 16 |  |  |
| 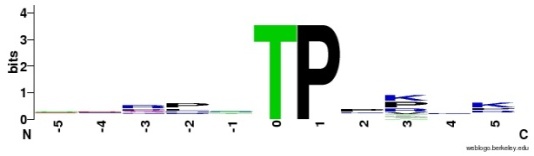 | 19 | ......TP..... | 19 |
