# Supplementary material for: Identifying Protein Phosphorylation Sites with Kinase Substrate Specificity on Human Viruses
Source: PLoS One. 2012 Jul 23;7(7):e40694. doi: 10.1371/journal.pone.0040694 (PMC3402495; doi:10.1371/journal.pone.0040694)
Supplement: Table S8 — The amino acids group used in MDD clustering. (DOCX) [file pone.0040694.s010.docx]

**Supplementary Table S8.** The amino acids group used in MDD clustering.

| **Group name** | **Amino acids** |
| --- | --- |
| **Neutral** | Threonine (T), valine (V), leucine (L), isoleucine (I), methionine (M), glycine (G), alanine (A), serine (S), cysteine (C) |
| **Acid** | Aspartic acid (D), asparagine (N), glutamic acid (E), glutamine (Q) |
| **Basic** | Lysine (K), arginine (R), histidine (H) |
| **Aromatic** | Phenylalanine (F), tyrosine (Y), tryptophan (W) |
| **Imino** | Proline (P) |
