# Supplementary material for: Identifying Protein Phosphorylation Sites with Kinase Substrate Specificity on Human Viruses
Source: PLoS One. 2012 Jul 23;7(7):e40694. doi: 10.1371/journal.pone.0040694 (PMC3402495; doi:10.1371/journal.pone.0040694)
Supplement: Table S9 — Summary of Human Viruses. (DOCX) [file pone.0040694.s011.docx]

**Supplementary Table S9**. Summary of Human Viruses

| **Virus Name** | **Abbreviation** |
| --- | --- |
| Human Herpes Virus | HHV |
| Human Immunodeficiency Virus | HIV |
| Human T-Lymphotrophic Virus | HTLV |
| Human Respiratory Syncytial Virus | HRSV |
| Human Papillomavirus | HPV |
| Hepatitis C Virus | HCV |
| Herpes Simplex Virus | HSV |
| Varicella-Zoster Virus | VZV |
