# Supplementary material for: Identifying Protein Phosphorylation Sites with Kinase Substrate Specificity on Human Viruses
Source: PLoS One. 2012 Jul 23;7(7):e40694. doi: 10.1371/journal.pone.0040694 (PMC3402495; doi:10.1371/journal.pone.0040694)
Supplement: Table S10 — Summary of Human Kinases. (DOCX) [file pone.0040694.s012.docx]

**Supplementary Table S10**. Summary of Human Kinases

| **Kinase Name** | **Abbreviation** |
| --- | --- |
| Caseine Kinase 2 | CK2 |
| Protein Kinase B | PKB |
| Cyclin-dependent Kinase | CDK |
| Mitogen-activated Protein Kinase | MAPK |
| Extracellular Signal-regulated Kinase | ERK |
